# Separate base usages of genes located on the leading and lagging strands in *Chlamydia muridarum *revealed by the Z curve method

**DOI:** 10.1186/1471-2164-8-366

**Published:** 2007-10-10

**Authors:** Feng-Biao Guo, Xiu-Juan Yu

**Affiliations:** 1 School of Life Science and Technology, University of Electronic Science and Technology of China, Chengdu, 610054, China; 2 Department of clinical medicine, West China Medical School, Sichuan University, Chengdu, 610091, China

## Abstract

**Background:**

The nucleotide compositional asymmetry between the leading and lagging strands in bacterial genomes has been the subject of intensive study in the past few years. It is interesting to mention that almost all bacterial genomes exhibit the same kind of base asymmetry. This work aims to investigate the strand biases in *Chlamydia muridarum *genome and show the potential of the Z curve method for quantitatively differentiating genes on the leading and lagging strands.

**Results:**

The occurrence frequencies of bases of protein-coding genes in *C. muridarum *genome were analyzed by the Z curve method. It was found that genes located on the two strands of replication have distinct base usages in *C. muridarum *genome. According to their positions in the 9-D space spanned by the variables *u*_1 _– *u*_9 _of the Z curve method, *K*-means clustering algorithm can assign about 94% of genes to the correct strands, which is a few percent higher than those correctly classified by *K*-means based on the RSCU. The base usage and codon usage analyses show that genes on the leading strand have more G than C and more T than A, particularly at the third codon position. For genes on the lagging strand the biases is reverse. The y component of the Z curves for the complete chromosome sequences show that the excess of G over C and T over A are more remarkable in *C. muridarum *genome than in other bacterial genomes without separating base and/or codon usages. Furthermore, for the genomes of *Borrelia burgdorferi*, *Treponema pallidum*, *Chlamydia muridarum *and *Chlamydia trachomatis*, in which distinct base and/or codon usages have been observed, closer phylogenetic distance is found compared with other bacterial genomes.

**Conclusion:**

The nature of the strand biases of base composition in *C. muridarum *is similar to that in most other bacterial genomes. However, the base composition asymmetry between the leading and lagging strands in *C. muridarum *is more significant than that in other bacteria. It's supposed that the remarkable strand biases of G/C and T/A are responsible for the appearance of separate base or codon usages in *C. muridarum*. On the other hand, the closer phylogenetic distance among the four bacterial genomes with separate base and/or codon usages is necessary rather than occasional. It's also shown that the Z curve method may be more sensitive than RSCU when being used to quantitatively analyze DNA sequences.

## Background

The compositional asymmetry between the leading and lagging strands in bacterial genomes has been the subject of intensive study in the past few years [1-11]. It is interesting that almost all bacterial genomes exhibit the same kind of asymmetry [3,5,8,9] i.e., there is an excess of nucleotides G relative to C in the leading strand and of C to G in the lagging strand, which is frequently accompanied by an abundance of T over A in the leading strand [3,8-11]. There is not a relationship between base composition biases and genomic G+C contents [1,3,8]. The excesses of G relative to C and T relative to A can be generally measured by GC skew and AT skew, which are given by (G-C)/(G+C) and (A-T)/A+T), respectively [1]. The GC skew (and AT skew) has (have) been used to map or relocate the replication origins of many bacteria such as *Mycoplasma genitalium *[12]*, Treponema pallidum *[13] and *Borrelia burgdoreferi *[14]. Several plausible explanations have been proposed that account for the biases in base composition, which have been partly summarized in four recent papers [5,7-9]. It seems that the cytosine deamination theory enjoys the most attention among the theories aimed at explaining strand biases [6,9]. The deamination of cytosine leads to the formation of uracil. In normal circumstance *in vivo*, cytosine is effectively protected against deamination because of the Watson-Crick base paring [6]. But the rate of cytosine deamination increases 140 times when the DNA is single-stranded [15,16]. If the resulting uracil is not replaced with cytosine, C to T mutation occurs. During the process of replication, the leading strand is more exposed in the single-stranded state [17]. Therefore, the C to T mutation occurs more frequently in the leading stand than in the lagging strand and the excesses of G(C) relative to C(G) and T(A) relative to A(T) are formed in the leading(lagging) strand.

The single base biases may propagate into higher-order biases in a correlated way, thereby changing the relative frequencies of codons and even amino acids of genes and encoded proteins in each of the replicating strands [9]. Salzberg et al. observed the oligomer skews in about a dozen of bacterial genomes [4]. Rocha et al. observed compositional asymmetry between the genes located on the leading and lagging strands at the level of codons and amino acids [18]. Mcinerney showed that genes in *B. burgdoreferi *have two significantly different codon usages, depending on whether the gene is transcribed on the leading or lagging strand of replication [19]. Strand-specific codon usage bias was not a new observation, but for the first time it could be shown that the codon usage of the genes in both strands of replication was separate [19]. Frank and Lobry suggested that the separate codon usages observed in *B. burgdoreferi *may be an exceptional case [6]. Interestingly, the separate codon usages of the genes transcribed on the two strands was also observed in *T. pallidum *and *C. trachomatis*, respectively [20,21].

The complete genome sequence of *Chlamydia muridarum *has been reported [22]. In this paper, we show that *C. muridarum *genes have two separate base usages (or nucleotide compositions at three codon positions) depending on whether the gene is transcribed on the leading or lagging strand, using the Z curve and CA methods (Different from [19-21], where CA of RSCU was carried out). There is also significant difference between the codon usage of the genes encoded on the two strands. The difference between the *y *component of the Z curves for *C. muridarum *and *E. coli *chromosome sequences may be advantageous to understand the appearance of the separate base usages in *C. muridarum*.

## Results and discussion

### (1) Strand-specific base usage biases revealed using CA of *u*_1 _– *u*_9_

**Figure 1:**
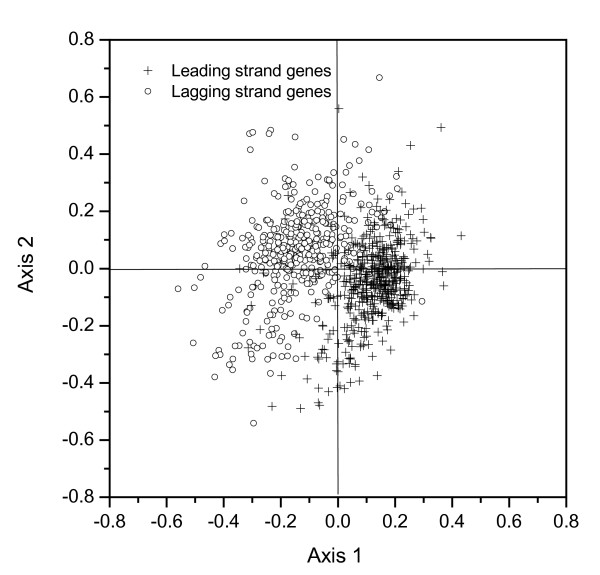
The distribution of points based on the two most important axes using the correspondence analysis of the nine variables *u*_1 _– *u*_9 _for 909 genes of the *C. muridarum *genome. The genes transcribed on the leading strand are denoted by crosses, whereas the genes located on the lagging strand are denoted by open circles. The partition between the two categories of points in the plot shows that the genes located on the two strands of replication have separate base usages.

For each of the 909 genes in *C. muridarum*, the nine variables *u*_1 _– *u*_9 _were calculated, which correspond to a point in a 9-D space. In order to visualize the distribution of mapping points in the 9-D space, project them onto a 2-D plane spanned by the first and second principal axes using the CA method. The first and second principal axes account for 28.0% and 23.8% of the total inertia of the 9-D space, respectively. And no other axes account for more than 12%. Figure 1 shows the position of the genes on the 2-D principal plane. As can be seen, all the genes are separated into two distinct clusters with little overlap, which indicate the genes in the two clusters have different base usage.

On inspection, it was found that the two groups correspond to the genes that are transcribed either in the leading strand or in the lagging strand, respectively. According to the locations of the origin and termination of replication determined using GC skew [22], 498 genes are located on the leading strand and the other 411 ones are located on the lagging strand. The genes transcribed in the leading and lagging strands are denoted by open crosses and open circles, respectively, in Figure 1. This phenomenon, i.e., separate base usages of the genes in the two strands of replication, is similar to the separate codon usages observed previously in *B. burgdorferi *[19], *T. pallidum *[20] and *C. trachomatis *[21], where CA of RSCU was used instead of CA of the variables *u*_1 _– *u*_9_. The base usage of the genes in the two strands is listed in Table 1. As we can see, the genes in the leading strand have a large excess of G to C and a little excess of T to A, particularly at the third codon position. While for the lagging strand, contrary case occurs. This observation agrees with the universal pattern of strand compositional asymmetry that appears in almost all the bacterial genomes. The cause of the strand compositional asymmetry is attributable to the disparity in the mutational bias (or the superimposition of a differential mutation rate and a differential correction/repair rate, as suggested by some researchers) between the leading and lagging strands. Among those theories aimed at explaining the strand biases, the cytosine deamination theory enjoys the most attention [6,9]. It should be noted that for the genes in the leading strand, the frequencies of G is less than that of C at the second codon position, which is contrary to the case at the other two codon positions. This may indicate the selection for efficient translation or possibly selection at the level of amino acid composition [23].

**Table 1:** Base usage for genes located on the leading and lagging strands in the *C. muridarum*

	Leading strand						Lagging strand					
	
	a	c	g	t	g-c	t-a	a	c	g	t	g-c	t-a
1^st ^codon position	0.261	0.177	0.333	0.229	0.156	-0.032	0.280	0.222	0.271	0.227	0.049	-0.053
2^nd ^codon position	0.303	0.208	0.172	0.316	-0.036	0.02	0.300	0.243	0.140	0.317	-0.103	0.017
3^rd ^codon position	0.284	0.122	0.212	0.383	0.090	0. 099	0.307	0.199	0.135	0.359	-0.0643	0.052
Average	0.283	0.169	0.239	0.309	0.070	0.029	0.296	0.221	0.182	0.301	-0.039	0.005

### (2) Using the *K*-means clustering of *u*_1 _– *u*_9 _to differentiate quantitatively genes in the two strands of replication

To differentiate quantitatively genes in the leading and lagging strands according to their positions in the 9-D space spanned by the variables *u*_1 _– *u*_9_, *K*-means clustering is employed based on the nine variables *u*_1 _– *u*_9_. It is obvious that the number of classes K is assigned to be 2. Consequently, 852 of the 909 *C. muridarum *genes are clustered into the right classes, i.e., about 94% of the genes have base usage typical of their own strand. The other 57 are wrongly clustered. Among them, 18 belong to leading strand and 39 belong to lagging strand. As mentioned above, in *B. burgdorferi*, *T. pallidum *and *C. trachomatis *genomes, separate codon usages of the genes in the two strands of replication has been observed by other researchers using CA of RSCU. Here we tackle this issue using the Z curve method. For each genome, the nine variables *u*_1 _– *u*_9 _are calculated for each gene. Then *K-means *clustering is employed based on *u*_1 _– *u*_9 _and the results are listed in Table 2. As we can see, for *B. burgdorferi*, *T. pallidum *and *C. trachomatis *genomes, 96.9%, 89.5% and 93.3% of the genes are assigned to the correct strand, respectively. From Table 2, it's also found that there are more genes located on the leading strand than in the lagging strand, which has been observed previously by many other researchers [3].

**Table 2:** The Results of *K*-means clustering based on the variables *u*_1_–*u*_9 _defined in equation (3)

	Leading strand		Lagging strand		Total ^a^	
	
	No. of genes	Clustered correctly^a^	No. of genes	Clustered correctly ^a^	No. of genes	Clustered correctly ^a^
*B. burgdoreferi*^b^	565	547 (96.8%)	285	277 (97.2%)	850	824 (96.9%)
*T. pallidum*^c^	680	604 (88.8%)	351	319 (90.9%)	1031	923 (89.5%)
*C. trachomatis*^d^	494	479 (97.0%)	400	355 (88.8%)	894	834 (93.3%)
*C. muridarum *^e^	499	481 (96.4%)	410	372 (90.7%)	909	853 (93.8%)

^a ^The percentage in the parenthesis denotes the number of the genes clustered correctly divided by the total number of the genes.

^b ^The origin of replication of the linear chromosome is assumed to be upstream of *dnaA *(BB0437 gene).

^c ^The origin and termination of replication are assumed to be upstream of *dnaA *(TP001 gene) and between genes TP0515 and TP0516, respectively.

^d ^The origin of replication is assumed to lie between CT632 and CT633, while the termination is assumed to lie between CT177 and CT178.

^e ^The locations of the origin and termination of replication have been mentioned in the Section Material and Method.

### (3) Codon usage bias

The individual base biases may propagate into higher-order biases in a correlated way, thereby changing the relative frequencies of codons and even amino acids of genes and encoded proteins in each of the replicating strands [9]. The cumulative codon usage for the genes located on the two strands of replication in *C. muridarum *is shown in Table 3. The total numbers of codons in the leading and lagging strands are 177130 and 146226 respectively. Chi-squared test of RSCU is carried out to evaluate the difference in codon usage between the two classes of genes. Significance is examined at the 5% level (*x*^2 ^value of 3.841). Among 59 codons, 54 are found to be significantly different in leading strand versus lagging strand genes. The codons are marked with << (the frequency in leading strand is larger than that in lagging strand) or >> (the frequency in lagging strand is larger than that in leadding strand). The 29 codons used more frequently in the leading strand are G-ending or T-ending, except TTA, ATA and AGA. Only one of the 25 codons used more frequently in the lagging strand is neither C-ending nor A-ending. The outlier is CTT.

**Table 3:** Codon usage for genes located on the leading and lagging strands in the *C. muridarum *genome

AA		Leading		Significant ^a^	Lagging	
				
		N	RSCU		N	RSCU
Phe	TTT	6116	1.45	>>	4543	1.24
	TTC	2333	0.55	<<	2806	0.76
Leu	TTA	6382	2.01	>>	4608	1.61
	TTG	4653	1.46	>>	1955	0.68
	CTT	3866	1.21	<<	4497	1.57
	CTC	1099	0.35	<<	2230	0.78
	CTA	1748	0.55	<<	2616	0.91
	CTG	1348	0.42	--	1266	0.44
Ile	ATT	6568	1.78	>>	5719	1.60
	ATC	2264	0.61	<<	3227	0.91
	ATA	2225	0.60	>>	1747	0.49
Met	ATG	3833	1.00	--	2565	1.00
Val	GTT	5637	1.73	>>	3056	1.59
	GTC	1470	0.45	<<	1515	0.79
	GTA	3172	0.97	<<	2012	1.05
	GTG	2766	0.85	>>	1104	0.57
Tyr	TAT	4137	1.52	>>	2842	1.27
	TAC	1321	0.48	<<	1628	0.73
TER	TAA	270	0.00	--	256	0.00
	TAG	161	0.00	--	89	0.00
His	CAT	2882	1.58	>>	2406	1.31
	CAC	776	0.42	<<	1276	0.69
Gln	CAA	4154	1.17	<<	4833	1.52
	CAG	2974	0.83	>>	1539	0.48
Asn	AAT	4480	1.52	>>	3811	1.29
	AAC	1418	0.48	<<	2085	0.71
Lys	AAA	6850	1.29	<<	6888	1.60
	AAG	3801	0.71	>>	1726	0.40
Asp	GAT	7033	1.66	>>	4364	1.43
	GAC	1462	0.34	<<	1758	0.57
Glu	GAA	7392	1.18	<<	6318	1.49
	GAG	5141	0.82	>>	2180	0.51
Ser	TCT	5851	2.58	--	5418	2.51
	TCC	1530	0.67	<<	2523	1.17
	TCA	1626	0.72	--	1640	0.76
	TCG	1193	0.53	>>	786	0.36
Pro	CCT	3633	2.16	>>	3588	1.99
	CCC	834	0.50	<<	1532	0.85
	CCA	1606	0.95	--	1648	0.91
	CCG	666	0.40	>>	454	0.25
Thr	ACT	2794	1.44	>>	2898	1.35
	ACC	1010	0.52	<<	1875	0.88
	ACA	2504	1.29	--	2849	1.33
	ACG	1466	0.75	>>	939	0.44
Ala	GCT	6448	2.01	>>	4661	1.82
	GCC	1459	0.45	<<	1797	0.70
	GCA	3191	0.99	<<	2826	1.11
	GCG	1753	0.55	>>	936	0.37
Cys	TGT	2044	1.41	>>	1266	1.08
	TGC	851	0.59	<<	1079	0.92
TER	TGA	69	0.00	--	65	0.00
Trp	TGG	1863	1.00	--	1275	1.00
Arg	CGT	2571	1.65	>>	1399	1.40
	CGC	915	0.59	<<	1351	1.35
	CGA	1844	1.18	<<	1362	1.36
	CGG	989	0.63	>>	336	0.34
Ser	AGT	2208	0.97	>>	1149	0.53
	AGC	1211	0.53	<<	1411	0.65
Arg	AGA	2228	1.43	>>	1284	1.28
	AGG	815	0.52	>>	271	0.27
Gly	GGT	2331	0.76	>>	1199	0.59
	GGC	1241	0.41	<<	1312	0.64
	GGA	5116	1.67	<<	4021	1.98
	GGG	3538	1.16	>>	1611	0.79

^a ^Results of Chi-squared test: << indicates that the leading strand genes used the condon more frequently than the lagging strand genes; >> indicates the lagging strand genes used the codon more frequently than the leading strand genes; -- indicates that there is no significant difference in usage of the codon on either strand. Significance is examined at the level of 5%.

If *K-means *clustering is applied based on RSCU, then 842(842/909 = 92.6%) *C. muridarum *genes will be assigned to the correct strands. The ratio is a little lower than using the variables *u*_1 _– *u*_9 _of the Z curve method. Similar result is observed for *B. burgdorferi*, *T. pallidum *and *C. trachomatis *genomes. This may indicate that the Z curve method is more sensitive than RSCU when being used to quantitatively analyze the DNA sequences.

### (4) Why do the separate base usages (or codon usages) appear in some special genomes while not in other genomes?

To our knowledge, the similar phenomenon, separate base usages or codon usages of the genes located on the leading and lagging strands has been observed in four bacterial genomes, *B. burgdorferi*, *T. pallidum*, *C. trachomatis *and *C. muridarum*. In this section, we do not focus on the underlying mechanism of the strand mutation biases, which lead to the compositional asymmetry in turn. We only want to give a possible clue about why the separate codon or base usage appears in special genomes and not in other genomes. The nature of the strand biases at the level of base composition is the same in almost all the bacterial genomes. But only several genomes have been reported of the separate base or codon usages. These four species have very different genome G+C contents, respectively, 28.6% (*Borrelia burgdorferi*), 40.3% (*Chlamydia muridarum*),41.3% (*Chlamydia trachomatis*) and 52.8%(*Treponema pallidum*). The common features of these four genomes are discussed in the following.

**Figure 2:**
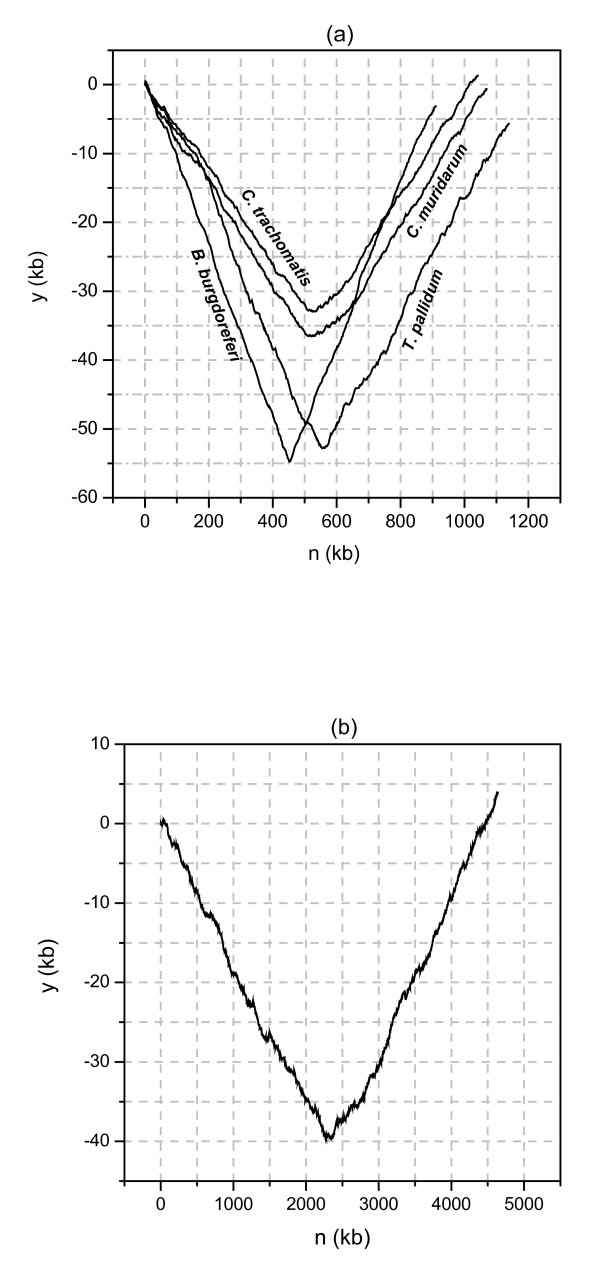
(a) The y components of the Z curves for *B. burgdorferi*, *T. pallidum*, *C. trachomatis *and *C. muridarum *genomes. (b) The y component of the Z curve for *E. coli *K-12 genome. Comparing (a) with (b) and putting emphasis on the coordinate values, it is found that the y component increases or decreases much slower along the DNA sequence for *E. coli genome *than that for the other four genomes. Note that the y component of the Z curve represents the plus of cumulative excess of G over C and T over A. Therefore, there are remarkable excess of G over C and T over A in the four bacterial genomes.

Firstly, from the genome sequence, perhaps we can find some clues related to the appearance of the separate codon or base usage of the genes in the two strands of replication. To achieve this aim, the y components of the Z curves of the complete chromosme sequences for *Borrelia burgdorferi*, *Chlamydia muridarum*, *Chlamydia trachomatis*, *Treponema pallidum *and *E. coli *K12 are investigated. According to equation (1), the y component curve represents the plus of cumulative excess of G over C and T over A. Due to this feature, the y component curve has been used to successfully map the replication origin and termination for a recently sequenced archaea *Methanoasarcina mazei *[24]. To allow convenient observation and direct comparison among different genomes, the first base of the chromosome sequence is shifted to the origin of replication for each genome in this study. In Figure 2(a), the y component curves for four genomes with separate codon or base usage being observed are shown. On the other hand, the y component curve for *E. coli *K12 genome is shown in Figure 2(b). The separate base or codon usages of the genes in the two strands of replication is not observed in *E. coli *K12, though the G/C and T/A strand composition biases exist, too. Both the curves in Figure 2(a) and in Figure 2(b) are linear symmetry, which shows the composition variation along the leading and lagging strands are basically complementary. Consequently, only the left half of each curve studied is adequate. The left half of each curve is an approximate line with steady climb along the DNA sequence. Let *k *denote the variation rate of y component with the increase of base step. In fact, *k *is equal to (*G-C+T-A*)/(*G+C+T+A*). Roughly calculating from Figure 2, the *k *values for two spirochetes (*B. burgdorferi *and *T. pallidum*) and two Chlamydiae (*C. muridarum *and *C. trachomatis*) are about 0.1 and 0.07, respectively. However, the value of *k *for *E. coli *K-12 is less than 0.02. Generally speaking, the *k *values for other bacterial genomes are nearly equal to or less than that for *E. coli *K-12 genome. The difference of the *k *values among the four genomes and *E. coli *K-12 indicates that there are much more GC and TA strand biases in the chromosome sequences in the four bacteria than in other species. Then it would be reasonable to propose that the remarkable strand biases of GC and TA cause the appearance of separate codon or base usage in special bacterial genomes.

Furthermore, from the phylogenetic point of view, the four genomes have the closer phylogenetic distance than that with other class (or order). *Borrelia burgdorferi *and *Treponema pallidum *belong to *spirochaetes *family. On the other hand, *Chlamydia muridarum *and *Chlamydia trachomatis *belong to *Chlamydia *genera. According to Wolf et al. [25] and Qi et al. [26], *Spirochetes *and *chlamydiae *are grouped together in the evolutional tree. We would like to believe that the closer phylogenetic distance among the four genomes is necessary rather than occasional.

## Conclusion

It's shown that protein-coding genes in *C. muridarum *genome have two separate and significantly different base (and codon) usages, depending on whether the gene is transcribed on the leading or lagging strand of replication. According to their positions in the 9-D space spanned by the variables *u*_1 _– *u*_9 _of the Z curve method, *K*-means clustering algorithm can classify about 94% of the genes into the correct strands, which is a few percent higher than those correctly classified by *K*-means of RSCU. The remarkable strand biases of G/C and T/A are supposed to be responsible for the appearance of separate base or codon usages in *C. muridarum*. Furthermore, *B. burgdorferi*, *T. pallidum*, *C. muridarum *and *C. trachomatis *have closer phlygenetic distance than that with other class (or order), in which the separate base and/or codon usages have been observed.

## Methods

### The database

The *C. muridarum *genome DNA sequence and the annotation information were downloaded from GenBank ftp site [27]. 909 protein-coding genes are listed in the annotation. GC skew with a non-overlapping sliding window of 1000 bp was used to determine the origin and termination of replication, as described by Read et al. [22]. Consequently, the origin was assumed to lie before gene TC0001, whereas the termination between genes TC0438 and TC0439.

### The Z curve

The Z curve is a 3-D space curve constituting the unique representation of a given DNA sequence in the sense that for the curve and sequence each can be uniquely reconstructed from the other [28]. Denoting the cumulative occurring numbers of the bases A, C, G and T in a DNA sequence read from the 5' to the 3'-end by A_*n*_, C_*n*_, G_*n *_and T_*n*_, respectively, we defined the Z curve in the following. The Z curve consists of a series of nodes P_*n*_, where *n *= 1, 2, ..., *N*, whose coordinates are denoted by *x*_*n*_, *y*_*n *_and *z*_*n*_. It was shown [28] that:

xn=(An+Gn)−(Cn+Tn)≡Rn−Ynyn=(An+Cn)−(Gn+Tn)≡Mn−Knzn=(An+Tn)−(Cn+Gn)≡Wn−Snn=0,1,2,...,N,xn,yn,zn∈[−N,N],
 MathType@MTEF@5@5@+=feaafiart1ev1aaatCvAUfKttLearuWrP9MDH5MBPbIqV92AaeXatLxBI9gBaebbnrfifHhDYfgasaacH8akY=wiFfYdH8Gipec8Eeeu0xXdbba9frFj0=OqFfea0dXdd9vqai=hGuQ8kuc9pgc9s8qqaq=dirpe0xb9q8qiLsFr0=vr0=vr0dc8meaabaqaciaacaGaaeqabaqabeGadaaakeaafaqaaeabdaaaaeaacqWG4baEdaWgaaWcbaGaemOBa4gabeaaaOqaaiabg2da9aqaaiabcIcaOiabbgeabnaaBaaaleaacqWGUbGBaeqaaOGaey4kaSIaee4raC0aaSbaaSqaaiabd6gaUbqabaGccqGGPaqkcqGHsislcqGGOaakcqqGdbWqdaWgaaWcbaGaemOBa4gabeaakiabgUcaRiabbsfaunaaBaaaleaacqWGUbGBaeqaaOGaeiykaKIaeyyyIORaeeOuai1aaSbaaSqaaiabd6gaUbqabaGccqGHsislcqqGzbqwdaWgaaWcbaGaemOBa4gabeaaaOqaaiabdMha5naaBaaaleaacqWGUbGBaeqaaaGcbaGaeyypa0dabaGaeiikaGIaeeyqae0aaSbaaSqaaiabd6gaUbqabaGccqGHRaWkcqqGdbWqdaWgaaWcbaGaemOBa4gabeaakiabcMcaPiabgkHiTiabcIcaOiabbEeahnaaBaaaleaacqWGUbGBaeqaaOGaey4kaSIaeeivaq1aaSbaaSqaaiabd6gaUbqabaGccqGGPaqkcqGHHjIUcqqGnbqtdaWgaaWcbaGaemOBa4gabeaakiabgkHiTiabbUealnaaBaaaleaacqWGUbGBaeqaaaGcbaGaemOEaO3aaSbaaSqaaiabd6gaUbqabaaakeaacqGH9aqpaeaacqGGOaakcqqGbbqqdaWgaaWcbaGaemOBa4gabeaakiabgUcaRiabbsfaunaaBaaaleaacqWGUbGBaeqaaOGaeiykaKIaeyOeI0IaeiikaGIaee4qam0aaSbaaSqaaiabd6gaUbqabaGccqGHRaWkcqqGhbWrdaWgaaWcbaGaemOBa4gabeaakiabcMcaPiabggMi6kabbEfaxnaaBaaaleaacqWGUbGBaeqaaOGaeyOeI0Iaee4uam1aaSbaaSqaaiabd6gaUbqabaaakeaaaeaaaeaafaqabeqacaaabaGaemOBa4Maeyypa0JaeGimaaJaeiilaWIaeGymaeJaeiilaWIaeGOmaiJaeiilaWIaeiOla4IaeiOla4IaeiOla4IaeiilaWIaemOta4KaeiilaWcabaGaemiEaG3aaSbaaSqaaiabd6gaUbqabaGccqGGSaalcqWG5bqEdaWgaaWcbaGaemOBa4gabeaakiabcYcaSiabdQha6naaBaaaleaacqWGUbGBaeqaaOGaeyicI4Saei4waSLaeyOeI0IaemOta4KaeiilaWIaemOta4Kaeiyxa0LaeiilaWcaaaaaaaa@A5E9@

where A_0 _= C_0 _= G_0 _= T_0 _= 0 and hence *x*_0 _= *y*_0 _= *z*_0 _= 0. Here R, Y, M, K, W, and S represent the bases of puRine, pYrimidine, aMino, Keto, Weak hydrogen bonds and Strong hydrogen bonds, respectively, according to the Recommendation 1984 by the NC-IUB [29]. The connection of the nodes P_0 _(P_0 _= 0), P_1_, P_2_, ..., until P_*N *_one by one sequentially by straight lines is called the Z curve for the DNA sequences inspected.

The Z curve offers an intuitive and convenient approach to study DNA sequences. By viewing the Z curve, some overall and local features of the sequence can be detected in a perceivable way. Furthermore, the phase-specific Z curve derived from the Z curve can be used for studying the nucleotide compositions in DNA fragments or [30,31] distinguishing the coding regions from the non-coding ones [32-34].

### The phase-specific Z curve

Suppose that the occurrence frequencies of the bases A, C, G and T at the 1st, 2nd and 3rd codon positions in a gene are denoted by *a*_*i*_, *c*_*i*_, *g*_*i *_and *t*_*i*_, respectively, where *i *= 1, 2, 3. On the basis of the Z curve theory [28], *a*_*i*_, *c*_*i*_, *g*_*i *_and *t*_*i *_are mapped onto a point in a 3-D space V_i _with the coordinates

xi=(ai+gi)−(ci+ti),yi=(ai+ci)−(gi+ti),zi=(ai+ti)−(ci+gi),i=1,2,3,xi,yi,zi∈[−1,1].
 MathType@MTEF@5@5@+=feaafiart1ev1aaatCvAUfKttLearuWrP9MDH5MBPbIqV92AaeXatLxBI9gBaebbnrfifHhDYfgasaacH8akY=wiFfYdH8Gipec8Eeeu0xXdbba9frFj0=OqFfea0dXdd9vqai=hGuQ8kuc9pgc9s8qqaq=dirpe0xb9q8qiLsFr0=vr0=vr0dc8meaabaqaciaacaGaaeqabaqabeGadaaakeaafaqaaeabdaaaaeaacqWG4baEdaWgaaWcbaGaemyAaKgabeaaaOqaaiabg2da9aqaaiabcIcaOiabdggaHnaaBaaaleaacqWGPbqAaeqaaOGaey4kaSIaem4zaC2aaSbaaSqaaiabdMgaPbqabaGccqGGPaqkcqGHsislcqGGOaakcqWGJbWydaWgaaWcbaGaeeyAaKgabeaakiabgUcaRiabdsha0naaBaaaleaacqWGPbqAaeqaaOGaeiykaKIaeiilaWcabaGaemyEaK3aaSbaaSqaaiabdMgaPbqabaaakeaacqGH9aqpaeaacqGGOaakcqWGHbqydaWgaaWcbaGaemyAaKgabeaakiabgUcaRiabdogaJnaaBaaaleaacqWGPbqAaeqaaOGaeiykaKIaeyOeI0IaeiikaGIaem4zaC2aaSbaaSqaaiabbMgaPbqabaGccqGHRaWkcqWG0baDdaWgaaWcbaGaemyAaKgabeaakiabcMcaPiabcYcaSaqaaiabdQha6naaBaaaleaacqWGPbqAaeqaaaGcbaGaeyypa0dabaGaeiikaGIaemyyae2aaSbaaSqaaiabdMgaPbqabaGccqGHRaWkcqWG0baDdaWgaaWcbaGaemyAaKgabeaakiabcMcaPiabgkHiTiabcIcaOiabdogaJnaaBaaaleaacqqGPbqAaeqaaOGaey4kaSIaem4zaC2aaSbaaSqaaiabdMgaPbqabaGccqGGPaqkcqGGSaalaeaaaeaaaeaacqWGPbqAcqGH9aqpcqaIXaqmcqGGSaalcqaIYaGmcqGGSaalcqaIZaWmcqGGSaalcqWG4baEdaWgaaWcbaGaemyAaKgabeaakiabcYcaSiabdMha5naaBaaaleaacqWGPbqAaeqaaOGaeiilaWIaemOEaO3aaSbaaSqaaiabdMgaPbqabaGccqGHiiIZcqGGBbWwcqGHsislcqaIXaqmcqGGSaalcqaIXaqmcqGGDbqxcqGGUaGlaaaaaa@8C17@

Then, each gene may be represented by a point or a vector in a 9-D space V, where V = V_1 _⊕ V_2 _⊕ V_3_, here the symbol ⊕ denotes the direct-sum of two subspaces. The nine components *u*_1 _– *u*_9 _of the space V are defined as follows

u1=x1,u2=y1,u3=z1,u4=x2,u5=y2,u6=z2,u7=x3,u8=y3,u9=z3.
 MathType@MTEF@5@5@+=feaafiart1ev1aaatCvAUfKttLearuWrP9MDH5MBPbIqV92AaeXatLxBI9gBaebbnrfifHhDYfgasaacH8akY=wiFfYdH8Gipec8Eeeu0xXdbba9frFj0=OqFfea0dXdd9vqai=hGuQ8kuc9pgc9s8qqaq=dirpe0xb9q8qiLsFr0=vr0=vr0dc8meaabaqaciaacaGaaeqabaqabeGadaaakeaafaqabeWabaaabaGaemyDau3aaSbaaSqaaiabigdaXaqabaGccqGH9aqpcqWG4baEdaWgaaWcbaGaeGymaedabeaakiabcYcaSiabdwha1naaBaaaleaacqaIYaGmaeqaaOGaeyypa0JaemyEaK3aaSbaaSqaaiabigdaXaqabaGccqGGSaalcqWG1bqDdaWgaaWcbaGaeG4mamdabeaakiabg2da9iabdQha6naaBaaaleaacqaIXaqmaeqaaOGaeiilaWcabaGaemyDau3aaSbaaSqaaiabisda0aqabaGccqGH9aqpcqWG4baEdaWgaaWcbaGaeGOmaidabeaakiabcYcaSiabdwha1naaBaaaleaacqaI1aqnaeqaaOGaeyypa0JaemyEaK3aaSbaaSqaaiabikdaYaqabaGccqGGSaalcqWG1bqDdaWgaaWcbaGaeGOnaydabeaakiabg2da9iabdQha6naaBaaaleaacqaIYaGmaeqaaOGaeiilaWcabaGaemyDau3aaSbaaSqaaiabiEda3aqabaGccqGH9aqpcqWG4baEdaWgaaWcbaGaeG4mamdabeaakiabcYcaSiabdwha1naaBaaaleaacqaI4aaoaeqaaOGaeyypa0JaemyEaK3aaSbaaSqaaiabiodaZaqabaGccqGGSaalcqWG1bqDdaWgaaWcbaGaeGyoaKdabeaakiabg2da9iabdQha6naaBaaaleaacqaIZaWmaeqaaOGaeiOla4caaaaa@6D39@

It is obvious that the nine variables *u*_1 _– *u*_9 _represent the base usage for a gene.

### Correspondence analysis

**Correspondence analysis **(CA) is a classical technique to reduce the dimensionality of the dataset by transforming to a new set of variables (the principal components) to summarize the feature of the data [35]. The new set of variables is derived from the linear combination of the original variables. The first principal axis is chosen to maximize the standard deviation of the derived variable and the second principal axis is the direction to maximize the standard deviation among directions un-correlated with the first, and so forth. For details about this method, refer to [36].

### *K*-means Clustering method

*K*-means clustering method [37] is adopted to differentiate quantitatively the leading and lagging strand genes according to their positions in the 9-D space (spanned by the variables *u*_1 _– *u*_9_) and 59-D space (spanned by RSCU values of codons, excluding three stop codons and the codons encoding for Met and Trp). *K*-means is a statistical method used to cluster data set into the given *K *classes based on the similarity of the elements. The idea in this method is to find a clustering (or grouping) of the observations so as to minimize the total within-cluster sums of squares. In this case, it sequentially processes each observation and reassigns it to another cluster if doing so results in a decrease in the total within-cluster sums of squares (referring to [37] for the details).

## Competing interests

The author(s) declares that there are no competing interests.

## Authors' contributions

FBG designed the work, wrote the computer program and the manuscript. XJY downloaded the data and performed the analysis. All authors read and approved the final manuscript.
